# Supplementary material for: Adiponectin receptor agonist AdipoRon improves skeletal muscle function in aged mice
Source: eLife. 2022 Mar 17;11:e71282. doi: 10.7554/eLife.71282 (PMC8963882; doi:10.7554/eLife.71282)
Supplement: Supplementary file 1. [file elife-71282-supp1.docx]

Supplement Table 1. List of primers used for real time PCR analysis

| **Gene name** | **Forward Primer** | **Reverse Primer** |
| --- | --- | --- |
| **Mouse** | | |
| *Cpt1a* | GAACCCCAACATCCCCAAAC | TCCTGGCATTCTCCTGGAAT |
| *Acadm* | ACACTCGAAAGCGGCTCACA | GCGGGCAGTTGCTTGAAC |
| *Acox1* | CAGGAAGAGCAAGGAAGTGG | CCTTTCTGGCTGATCCCATA |
| *Adipor1* | CCCCCTTACCCCCGTCCTTAC | GGCGTGGCTTTGTTTGTCCTA |
| *Adipor2* | TGCGCACACGTTTCAGTCTCCT | TTCTATGATCCCCAAAAGTGTGC |
| *Ppargc1a* | AGCCGTGACCACTGACAACGAG | GCTGCATGGTTCTGAGTGCTAAG |
| *Ppargc1a-1* | GGACATGTGCAGCCAAGACTCT | CACTTCAATCCACCCAGAAAGCT |
| *Ppargc1a-2* | CCACCAGAATGAGTGACATGGA | GTTCAGCAAGATCTGGGCAAA |
| *Ppargc1a-3* | AAGTGAGTAACCGGAGGCATTC | TTCAGGAAGATCTGGGCAAAGA |
| *Ppargc1a-4* | TCACACCAAACCCACAGAAA | CTGGAAGATATGGCACAT |
| *Myh7 (MyhcI)* | ACTGTCAACACTAAGAGGGTCA | TGCCCCAAAATGGATTCGGAT |
| *Myh2 (MyhcIIa)* | AAGTGACTGTGAAAACAGAAGCA | GCAGCCATTTGTAAGGGTTGAC |
| *Myh4 (MyhcIIb)* | CTTTGCTTACGTCAGTCAAGGT | AGCGCCTGTGAGCTTGTAAA |
| *Myh1 (MyhcIIx)* | CTCCAGGCTGCTTTAGAGGAA | CCTGCTCCTAATCTCAGCATCC |
| *Rn18s* | GTAACCCGTTGAACCCCATT | CCATCCAATCGGTAGTAGCG |
| *Actb* | GGCTGTATTCCCCTCCAT | CCAGTTGGTAACAATGCC |
| **Non-human primates** | | |
| *Cpt1a* | TCCAGTTGGCTTATCGTGGTG | TCCAGAGTCCGATTGATTTTTGC |
| *Acadm* | AGTTACTTATAGAGCACCAAGCGA | TTCTAAGCCCACTTTTCTGTCAT |
| *Acox1* | ATGCCCAAGTGAAGATCCAG | GAAGATGAGGGAGTTTGGCA |
| *Ppargc1a* | CTGCTAGCAAGTTTGCCTCA | AGTGGTGCAGTGACCAATCA |
| *Actb* | GTTGTCGACGACGAGCG | GCACAGAGCCTCGCCTT |
| *Rn18s* | AAGACGGACCAGAGCGAAAG | CAAATTAAGCCGCAGGCTCC |
